# A DFT Calculation of Fluoride-Doped TiO_2_ Nanotubes for Detecting SF_6_ Decomposition Components

**DOI:** 10.3390/s17081907

**Published:** 2017-08-18

**Authors:** Xiaoxing Zhang, Jun Zhang, Xingchen Dong, Hao Cui

**Affiliations:** 1 State Key Laboratory of Power Transmission Equipment & System Security and New Technology, Chongqing University, Chongqing 400044, China; junz2016@cqu.edu.cn (J.Z.); dxc_cqu@outlook.com (X.D.); cuihaocqu@163.com (H.C.); 2 School of Electrical Engineering, Wuhan University, Wuhan 430072, China

**Keywords:** SF_6_ decomposition component, fluorine-doped TiO_2_, first-principle simulation

## Abstract

Gas insulated switchgear (GIS) plays an important role in the transmission and distribution of electric energy. Detecting and analyzing the decomposed components of SF_6_ is one of the important methods to realize the on-line monitoring of GIS equipment. In this paper, considering the performance limits of intrinsic TiO_2_ nanotube gas sensor, the adsorption process of H_2_S, SO_2_, SOF_2_ and SO_2_F_2_ on fluoride-doped TiO_2_ crystal plane was simulated by the first-principle method. The adsorption mechanism of these SF_6_ decomposition components on fluorine-doped TiO_2_ crystal plane was analyzed from a micro perspective. Calculation results indicate that the order of adsorption effect of four SF_6_ decomposition components on fluoride-doped TiO_2_ crystal plane is H_2_S > SO_2_ > SOF_2_ > SO_2_F_2_. Compared with the adsorption results of intrinsic anatase TiO_2_ (101) perfect crystal plane, fluorine doping can obviously enhance the adsorption ability of TiO_2_ (101) crystal plane. Fluorine-doped TiO_2_ can effectively distinguish and detect the SF_6_ decomposition components based on theoretical analysis.

## 1. Introduction

Gas insulated switchgear (GIS), with benefits such as small land cover, flexible configuration, high safety, and high reliability, has been widely used in the power system [1]. However, during the long-term operation of GIS equipment, the internal insulation defects may cause partial discharge (PD). SF_6_ insulating gas in the GIS will decompose into some types of gases like SO_2_, H_2_S, SOF_2_, SO_2_F_2_ [2,3,4,5] under the effect of PD. With the decomposition of SF_6_, aging of GIS equipment and corrosion of metal surface will be accelerated, which ultimately may lead to breakdown of GIS equipment, affecting the stable operation of the power system [6]. Therefore, it is of great importance to detect the SF_6_ decomposition components in GIS equipment. On one hand, the type of PD can be confirmed by the types of decomposition components; on the other hand, by measuring the contents of decomposition, the level of PD can be determined, even the aging degree of GIS equipment. Therefore, through the monitoring of decomposition components, unnecessary losses can be reduced by timely taking precaution and preventing the breakdown of GIS equipment.

At present, utilizing the gas sensor method to achieve the goal of detecting SF_6_ decomposition components has advantages of a small detection unit, easy installation process, fast detection speed, and so on. Therefore, it is of great importance to study gas sensitive response of the gas sensors made of different gas-sensing materials. Our team studied the gas-sensing materials such as carbon nanotubes and graphene [7,8,9,10,11,12]. However, it is in the early stage of research, and further research needs to be conducted. At the same time, with the technology of TiO_2_ nanotube preparation becoming mature, the TiO_2_ nanotube gas sensor has advantages of high specific surface area and high symmetry, which makes it a research hotspot in the gas detection field. However, the inherent energy gap of TiO_2_ is comparatively large (more than 3.0 eV), which hinders its wide development. Research shows that the surface of TiO_2_ nanotubes can be modified by nonmetallic doping to improve its photosensitive, photocatalytic and other properties. Varghese et al [13,14] sputtered a layer of 10 nm thick of Pd on the surface of TiO_2_ nanotubes by thermal evaporation, which can improve the sensitivity of TiO_2_ nanotube gas sensor, and reduce the recovery time. More importantly, the improved sensor can detect H_2_ at room temperature. At present, the study of nitrogen doping in nonmetallic doping has received the most attention [15,16,17,18,19]. The results show that nitrogen doping can reduce the energy gap of TiO_2_, which makes the electrons in the valence band transition more easily. In addition, many studies have reported that TiO_2_ is modified by fluorine doping [20,21,22,23]. Fluorine doping does not basically change the size of the TiO_2_ energy gap, but it can promote the generation of oxygen hole defects, increase the surface acidity and Ti^3+^, which is beneficial to reducing the recombination rate of electron hole pairs, and thereby improving the photocatalytic activity [24].

With the successful doping of fluorine onto the surface of TiO_2_ [20,25,26], the study of application of fluorine-doped TiO_2_ nanomaterials in gas sensing detection is deficient. In this paper, the idea of using fluorine-doped TiO_2_ nanotubes gas sensor to detect SF_6_ decomposition components was determined. The adsorption process of H_2_S, SO_2_, SOF_2_ and SO_2_F_2_ gas molecules onto fluorine-doped anatase TiO_2_ (101) perfect crystal plane by first-principle calculation. The calculation parameters include adsorption energy, adsorption distance, charge transfer amount and the density of states. The simulation analysis can provide insight for the practical explanation of fluorine-doped TiO_2_ nanometer array gas sensor detecting SF_6_ decomposition gases from the microscopic point of view. Finally, the adsorption results of SO_2_, SOF_2_ and SO_2_F_2_ under different doping conditions were compared in this paper.

## 2. Calculation Parameters and Methods

**Figure 1:**
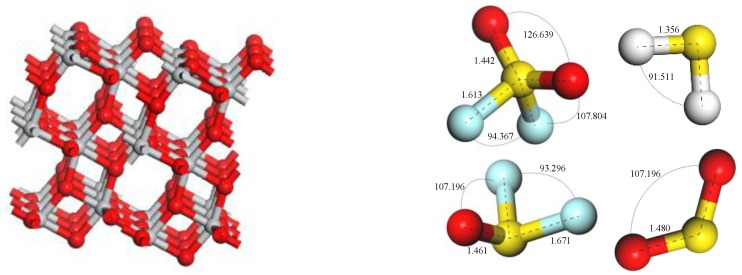
Perfect crystal model of intrinsic anatase TiO_2_ (101) and SO_2_, H_2_S, SOF_2_ and SO_2_F_2_ gas molecular models, Ti atom is gray, O atom is red, S atom is yellow, F atom is blue, H atom is white.

**Figure 2:**
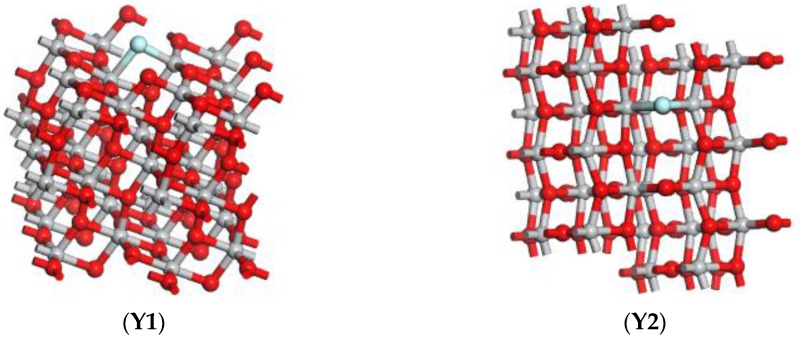
F-doped TiO_2_ model, (**Y1**) is the main view of the model, and (**Y2**) is the top view of the model.

The TiO_2_ model in this paper is an anatase TiO_2_ (101) perfect facet model derived directly from the database provided by Materials Studio software, and the size is 3.776 × 3.776 × 9.486 Å, which is the smallest unit of anatase TiO_2_. The detailed calculation process is as follows: build the anatase TiO_2_ (101) perfect crystal plane 2 × 2 super-cell model, and the gas molecules of SO_2_, H_2_S, SOF_2_ and SO_2_F_2_; optimize these models initially in the Dmol^3^ module, which can make these micro-structure parameters to maximumly close to the idealization, as shown in Figure 1.; replace one of the O atoms on the surface of the anatase TiO_2_ (101) perfect crystal plane by F atom; After optimized treatment, the fluorine doped anatase TiO_2_ (101) perfect crystal plane supercell model (F-doped TiO_2_) is obtained, as shown in Figure 2; finally, the optimized SO_2_, H_2_S, SOF_2_ and SO_2_F_2_ gas molecules with different postures approach respectively close to the surface of the perfect crystal plane of the anatase TiO_2_ (101) to get a different adsorption system. Different adsorption systems were optimized in order to find a more stable adsorption structure for each gas molecule onto F-doped TiO_2_.

In this paper, parameters are set as follows when optimizing the calculation. Since the number of atoms contained in the perfect crystal plane model of the anatase TiO_2_ (101) established in this paper is large, the generalized gradient approximate (GGA) with high calculation accuracy was adopted so as to make the calculated physical and chemical characteristics of the different adsorption system more accurate. The exchange and correlation interaction effect between electrons were represented by the Perdew-Burke-Ernzerhof (PBE) function [27]. The energy convergence tolerance and the energy gradient (max. force) are set to 1.0 × 10^−5^ Ha and 0.002 Ha/Å respectively. The atomic displacement (max. displacement) is set to 0.005 Å. The convergence accuracy of the self-consistent field charge density (SCF tolerance) is set to 1.0 × 10^−6^ Ha, Brillouin k-point grid (k-point) is set to 2 × 2 × 1; the double Numeric Basis with Polarization (DNP) was used in the atomic orbits calculation with d, p orbital polarization function at the same time so as to make the results more accurate. Considering the influence of the dispersion force, that is, the van der Waals force, the DFT-D (Grimme) algorithm was utilized; in order to improve the efficiency of computation, the direct inversion of iterative subspace (DIIS) is used to improve the convergence rate of the charge density of the self-consistent field.

## 3. Simulation Results and Analysis

### 3.1. Establishment of F-Doped TiO_2_ Model

The F-doped TiO_2_ model was established based on the perfect crystal plane model. A fluorine atom replaces one of the O atoms on the surface of the anatase TiO_2_ (101) perfect crystal plane, and F atom combined with Ti atoms to form Ti-F bonds.

**Figure 3:**
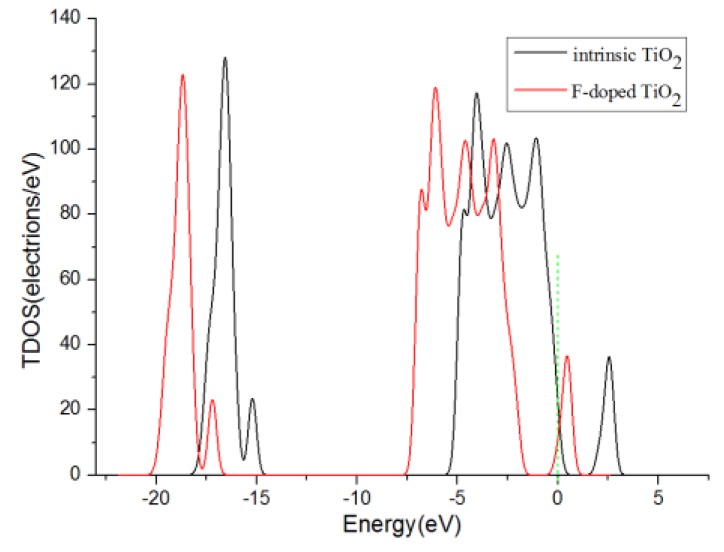
The curves of the density of states of F-doped TiO_2_ and intrinsic anatase TiO_2_ (101) perfect crystal plane, the green short dashed line is Fermi level.

Figure 3 shows the curve of the density of states (DOS) of F-doped TiO_2_ and intrinsic anatase TiO_2_ (101) perfect crystal plane. It can be seen from the figure that the peak and shape of the density curve almost did not change after the fluorine atom being doped. The doping of the fluorine atoms hardly changes the band gap of TiO_2_. However, after fluorine element doping, the Ti^4+^ is converted to Ti^3+^, and the presence of a certain amount of Ti^3+^ will reduce the recombination rate of electron hole pairs [28]. Furthermore, fluorine element doping is conducive to the generation of oxygen holes and enhances the mobility of effective electrons [24,29], which can enhance the conductivity of the adsorbent substrate and improve the gas sensing performance of the fluorine-doped TiO_2_ nano array gas sensor mentioned above.

### 3.2. Parameter Calculation of Different Adsorption Systems

**Figure 4:**
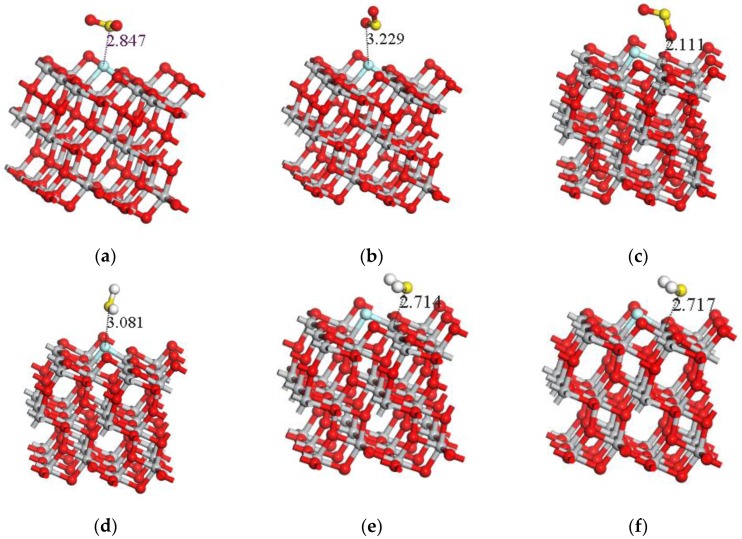

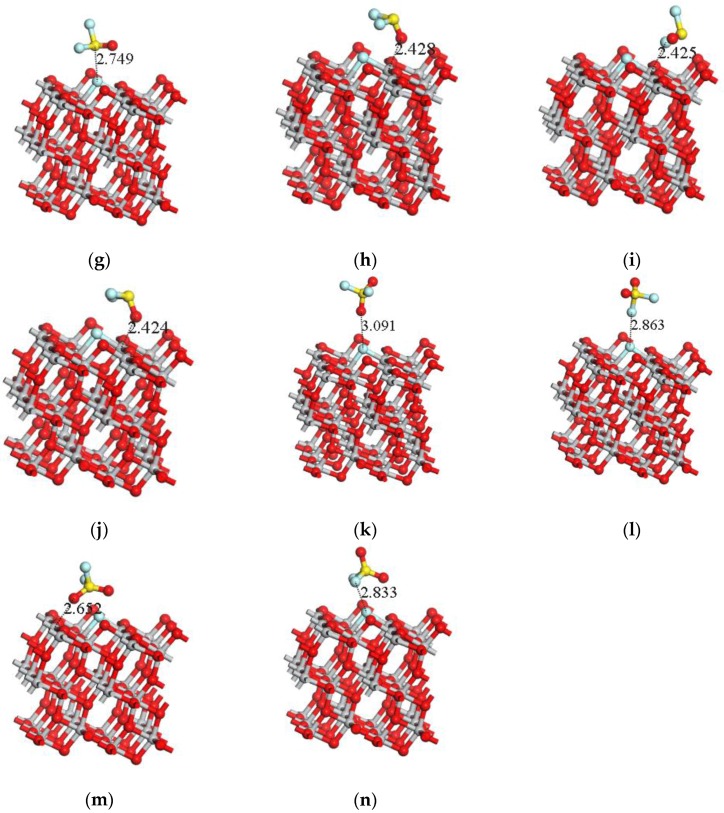
The adsorption structures of four gas molecules adsorbed on the crystal surface in different ways. (**a**–**n**) are the adsorption structure of four gas molecules adsorbed on the F-doped TiO_2_ crystal plane in different ways after complete optimization calculation.

Figure 4 shows the adsorption structure of four gas molecules adsorbed on the F atom of the F-doped TiO_2_ crystal planes in different ways after complete optimization calculation. Due to the structural characteristics of four gas molecules, the way that the gas molecule adsorbed on the crystal plane was considered. Three situations are considered when the SO_2_ molecule comes close to the crystal plane in the optimization calculations, that is a single S and O atoms close to the crystal plane and two O atoms simultaneously close to the crystal plane. And the adsorption structures after calculation are shown in Figure 4a–c. Three cases were considered for H_2_S molecules: the single S, H atoms close to the crystal plane and two H atoms simultaneously close to the crystal plane. The calculated adsorption structures are shown in Figure 4d–f. SOF_2_ molecules mainly consider four cases: the single S, O, F atoms close to the crystal plane and two F atoms simultaneously close to the crystal plane, the calculated adsorption structure are shown in Figure 4g–j; the SO_2_F_2_ molecule is of a tetrahedral structure, and the S atom is inside the structure. So, SO_2_F_2_ mainly considers 4 cases: a single O, F atom close to the crystal plane, two O atoms and two F atoms are simultaneously close to the crystal plane, separately. The calculated adsorption structures are shown in Figure 4k–n.

Adsorption energy is the degree of change of total energy before and after the adsorption of gas molecules onto crystal plane, which represents the ability of gas molecules adsorption onto the crystal plane. In this paper, the magnitude of adsorption energy is expressed by E_a_, and the formula is as follows:

E_a_ = E_sys_ − E_gas_ − E_sur_, (1)

where E_sys_ represents the energy of the whole system after the gas adsorbed on the F-doped TiO_2_, E_gas_ represents the energy when the gas molecules are present alone, and E_sur_ represents the energy of F-doped TiO_2_ without the adsorption of gas molecules.

In the adsorption process, besides the change of energy, it may also be accompanied with the electron transfer, which results in the change of the electronic structure of the crystal plane, and shows the changes of electrical properties such as resistance and capacitance at macro level. In practical applications, the gas sensitive response characteristics of gas sensors can be obtained by detecting these electrical characteristics. Therefore, this paper also calculated Mulliken charge distribution of the gas molecules adsorbed onto the F-doped TiO_2_, to confirm the amount of charge transfer before and after the adsorption of gas molecules onto the crystal plane. The charge transfer Q_t_ is defined as the charge change of gas molecules before and after they are adsorbed onto the F-doped TiO_2_ crystal plane. If Q_t_ > 0, the electron is transferred from the gas molecule to the crystal plane. On the contrary, if Q_t_ < 0, part of the electrons is transferred from the crystal plane to the gas molecule. Table 1 shows the adsorption energies, adsorption distance and charge transfer of four kinds of gas molecules SO_2_, H_2_S, SOF_2_ and SO_2_F_2_ adsorbed onto F-doped TiO_2_ crystal plane in different postures.

In Table 1, SO_2_-S-TiO_2_ represents an adsorption system in which SO_2_ molecules are close to the F-doped TiO_2_ crystal plane with single S atom, and others are similar. E_a_ < 0 indicates that the adsorption of four kinds of molecules onto F-doped TiO_2_ crystal plane are exothermic. The value of E_a_ is larger, indicates that the adsorption of gas molecules onto F-doped TiO_2_ crystal plane is easier, and adsorption structure is more stable.

When H_2_S gas molecules with a single H atom close to the crystal plane and two H atoms simultaneously close to crystal plane, the adsorption structure after optimization calculation (Figure 4e,f) is almost the same, the calculated adsorption energy (−0.837 eV and −0.836 eV) and the amount of charge transfer (0.267 e and 0.266 e) is almost exactly the same, which is larger than the adsorption energy and charge transfer (−0.209 eV and 0.008 e) of the adsorption with single S atom close to the crystal plane. At the same time, the adsorption distance (2.835 Å) of the adsorption system obtained from H_2_S gas molecules with single S atom close to the crystal plane should be larger than that of the other two approaching ways (2.714 Å and 2.717 Å). Therefore, the H_2_S gas molecules are much easier to adsorb onto the crystal plane with a single H atom close to the crystal plane and two H atoms simultaneously close to the F-doped TiO_2_ crystal plane. When the H_2_S gas molecules are adsorbed on the crystal plane, the amount of charge transferred to the crystal plane is about 0.266 e, and the macroscopic gas sensitivity of the gas sensor shows the decrease of the impedance.

Similarly, the adsorption reaction of SOF_2_ gas molecules more easily occurs in three cases: close to the F-doped TiO_2_ surface with a single O and F atoms and two F atoms at the same time, and the macroscopic sensitivity of the gas sensor shows the decrease of the impedance. SO_2_ gas molecule reacts easier with two O atoms at the same time when approaching the crystal surface, and the macroscopic gas sensitivity of the gas sensor shows the increase of the impedance. The comparison shows that the adsorption energy of H_2_S gas molecules onto F-doped TiO_2_ crystal plane is the highest, which is about two times of that of SOF_2_, SO_2_F_2_. In addition, when the three types of gas molecules, H_2_S, SOF_2_ and SO_2_F_2_, adsorb onto F-doped TiO_2_, the macroscopic gas sensitivity of the gas sensor shows the decrease of the impedance. When SO_2_ gas molecules react onto the F-doped TiO_2_, the macroscopic gas sensitivity of the gas sensor shows the increase of the impedance. Therefore, theoretically, F-doped TiO_2_ nanotube array gas sensor can effectively distinguish and detect the four kinds of gases.

### 3.3. Analysis of Density of States

When different gas molecules adsorb on the surface of the gas sensor, the resistance of the sensor may change; the fluorine-doped TiO_2_ nanotube array gas sensor mainly uses this principle to achieve the detection of SF_6_ decomposition components. So, one of the key parameters in practical applications is the resistance of the sensor and analysis of DOS of the adsorption system can be used to find the reasons from the change in resistance.

**Figure 5:**
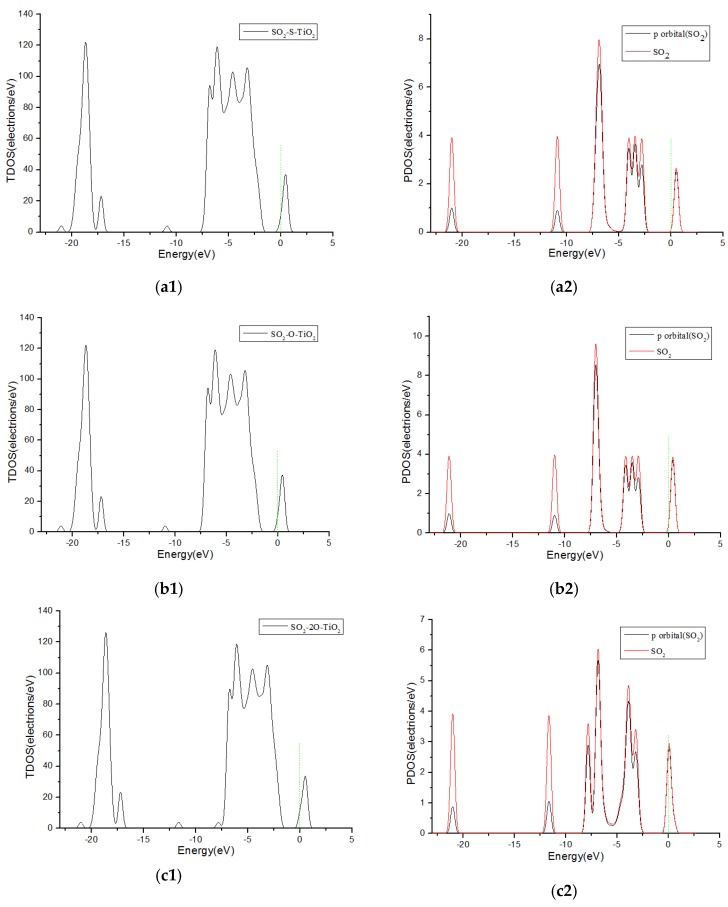
The total density and partial density of SO_2_ molecules adsorbed on F-doped TiO_2_, and the green short dashed line is Fermi level. (**a1**,**a2**) are the TDOS and PDOS of SO_2_-S-TiO_2_, (**b1**,**b2**) are the TDOS and PDOS of SO_2_-O-TiO_2_, (**c1**,**c2**) are the TDOS and PDOS of SO_2_-2O-TiO_2_.

Figure 5 shows the total density of states (TDOS) and the partial density of states (PDOS) curves of SO_2_ molecules adsorbed on F-doped TiO_2_. Since the contributes of p-orbit of the gas molecule is greatest to DOS, it is also presented in the figure. It can be seen from Figure 5(a1–b2), when the single S atom and the single O atom are close to the F-doped TiO_2_ crystal plane, the SO_2_ gas molecules contribute to the DOS of the adsorption system only on the right side of 0 eV. But, It can be seen from Figure 5(c1,c2), when the SO_2_ molecule close to the crystal plane with two O atoms at the same time, the SO_2_ molecules have a significant contribution to the DOS of the adsorption system on the both sides of 0 eV. This corresponds to the charge transfer amount (−0.013 e, 0 e and −0.12 e, respectively) when the SO_2_ molecules are adsorbed in three different ways in Table 1. It is shown that SO_2_ molecules are more likely to react and adsorb on the crystal plane approaching in the form of with two O atoms. The theoretical analysis shows that SO_2_ gas obtain electrons when adsorbed onto the fluorine-doped TiO_2_ nano sensors, and the macroscopic gas-sensing properties show an increase in impedance.

**Figure 6:**
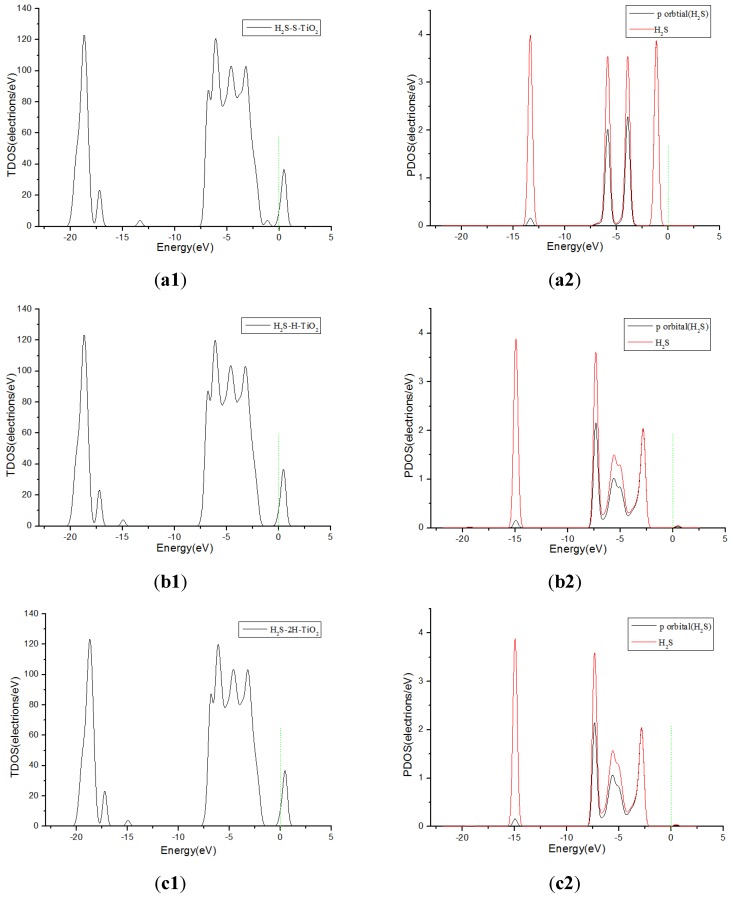
TDOS and PDOS of H_2_S molecules adsorbed onto F-doped TiO_2_, and the green short dashed line is Fermi level. (**a1**,**a2**) are the TDOS and PDOS of H_2_S-S-TiO_2_, (**b1**,**b2**) are the TDOS and PDOS of H_2_S-H-TiO_2_, (**c1**,**c2**) are the TDOS and PDOS of H_2_S-2H-TiO_2_.

**Figure 7:**
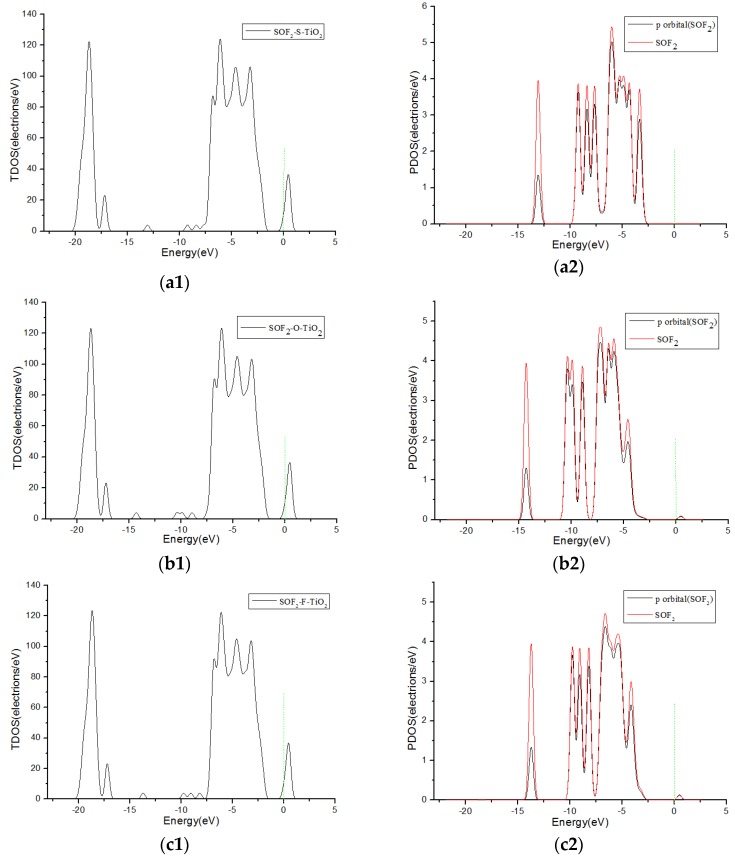

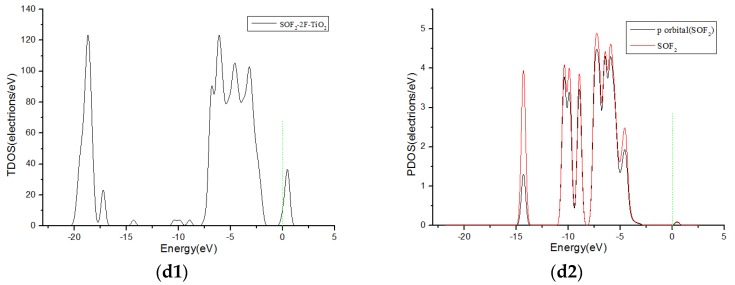
TDOS and PDOS of SOF_2_ molecules adsorbed onto F-doped TiO_2_, and the green short dashed line is Fermi level. (**a1**,**a2**) are the TDOS and PDOS of SOF_2_-S-TiO_2_, (**b1**,**b2**) are the TDOS and PDOS of SOF_2_-O-TiO_2_,(**c1**,**c2**) are the TDOS and PDOS of SOF_2_-F-TiO_2_, (**d1**,**d2**) are the TDOS and PDOS of SOF_2_-2F-TiO_2_.

Figure 6 shows TDOS and PDOS curves of H_2_S molecules adsorbed onto F-doped TiO_2_. When the H_2_S gas molecules close to the F-doped TiO_2_ crystal plane with single S atom, the gas molecules have little contribution to DOS at 0 eV in Figure 6(a1,a2). However, when H_2_S molecules approach the F-doped TiO_2_ crystal plane with single H atom and two H atoms at the same time separately, the molecules have a significant contribution to the DOS on the right side of 0 eV in Figure 6(b1–c2). This corresponds to the charge transfer amount (0.008 e, 0.267 e and 0.266 e, respectively) when the H_2_S molecule close to the crystal plane with single S atom, single H atom and two H atoms at the same time in Table 1. It is shown that H_2_S molecules are more likely to be adsorbed on the crystal plane approaching in the form of a single H atom and two H atoms at the same time. Similarly, SOF_2_ gas molecules are more likely to adsorb by single O, single F atom, and two F atoms at the same time when coming close to the crystal plane than by single S atom. The TDOS and PDOS curves of SOF_2_ adsorption systems are shown in Figure 7. The theoretical analysis shows that H_2_S and SOF_2_ gases lose electrons when adsorbed onto the fluorine-doped TiO_2_ nano sensors, and the macroscopic gas-sensing properties show a decrease in impedance. Furthermore, the changed value of impedance of H_2_S is bigger than SOF_2_.

**Figure 8:**
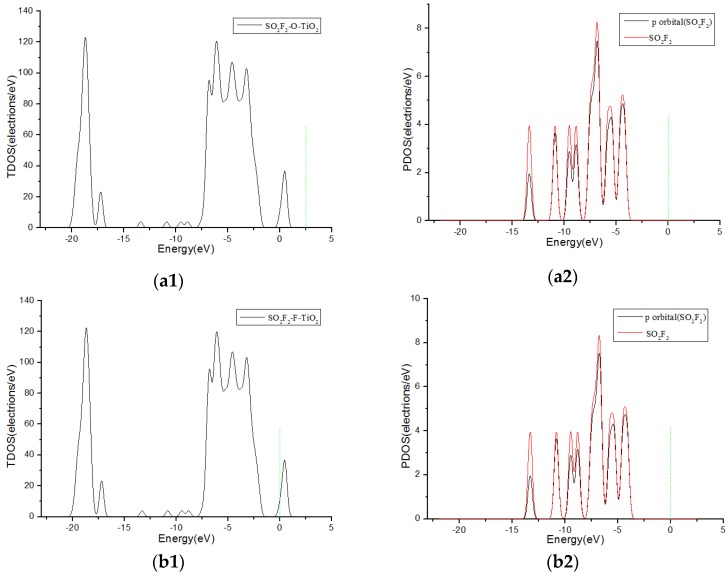

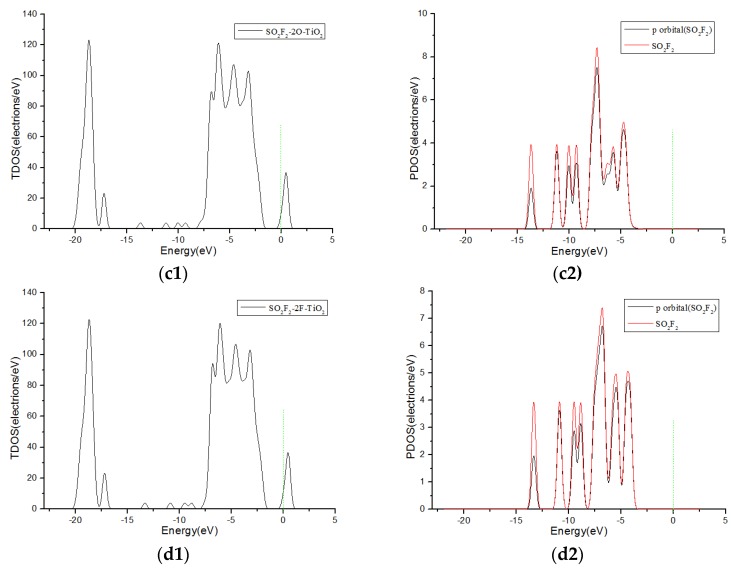
TDOS and PDOS of SO_2_F_2_ molecules adsorbed on F-doped TiO_2_, and the green short dashed line is Fermi level. (**a1**,**a2**) are the TDOS and PDOS of SO_2_F_2_-O-TiO_2_, (**b1**,**b2**) are the TDOS and PDOS of SO_2_F_2_-F-TiO_2_, (**c1**,**c2**) are the TDOS and PDOS of SO_2_F_2_-2O-TiO_2_, (**d1**,**d2**) are the TDOS and PDOS of SO_2_F_2_-2F-TiO_2_.

No matter which way the SO_2_F_2_ gas molecules approaching the crystal surface, the contribution to the DOS is almost 0 on both sides of 0eV, as shown in Figure 8. It can also be seen from Table 1 that the charge transfer is very small when SO_2_F_2_ molecules come close to the F-doped TiO_2_ crystal plane. The theoretical analysis indicates that the changed value of impedance of SO_2_F_2_ is small on the macro level when adsorbed onto the fluorine-doped TiO_2_ nano sensors. It can be assumed that the selectivity of fluorine-doped TiO_2_ nanotubes gas sensor to SO_2_F_2_ gas is weak.

### 3.4. Comparison of Adsorption Results under Different Doping Conditions

The reference [30] shows the adsorption results of SO_2_, SOF_2_ and SO_2_F_2_ onto the intrinsic anatase TiO_2_ (101) perfect crystal plane. By comparison, the adsorption structure of the three kinds of gas molecules adsorbed on the intrinsic anatase TiO_2_ (101) perfect crystal plane in the reference is similar to that of Figure 4c,h,m**.** Therefore, in order to make the comparison results more meaningful, adsorption parameters of adsorption structure in Figure 4c,h,m were chosen to be compared.

After fluorine doping on the anatase TiO_2_ (101) perfect crystal plane, the adsorption energy and charge transfer amount of the SO_2_, SOF_2_ and SO_2_F_2_ gas molecules adsorption system obviously increased, and the adsorption distance also decreased correspondingly. In addition, the adsorption energy of SO_2_ gas molecules adsorbed on F-doped TiO_2_ crystal plane is −0.617 eV, which indicates that the adsorption of SO_2_ gas molecules onto the F-doped TiO_2_ crystal plane was chemical adsorption.

The DOS were also compared and analyzed. In this paper, the corresponding TDOS and PDOS of the adsorption structures of three gas molecules are shown in Figure 5(c1,c2), Figure 7(b1,b2) and Figure 8(c1,c2). It was found that the contribution of SO_2_ and SOF_2_ molecules to the PDOS near 0eV significantly increased after fluorine doping, which corresponded to the charge transfer of the adsorption systems in the two cases. Because the crystal shows weak adsorption reaction to SO_2_F_2_ gas molecules before and after doping, the contribution of SO_2_F_2_ gas molecules to PDOS did not changed much at 0 eV. Through the above analysis, it can be concluded that the adsorption ability of the anatase TiO_2_ (101) crystal plane to the three gas molecules is enhanced after fluorine doping.

Reference [31] reported modified TiO_2_ crystal plane by nitrogen doping to detect SF_6_ decomposition components. It can be seen that SO_2_ molecules are more likely to adsorb on nitrogen-doped TiO_2_ crystal plane with S atom. SOF_2_ is more likely to adsorb on nitrogen-doped TiO_2_ crystal plane with O and S atoms, respectively. The adsorption of SO_2_ and SOF_2_ on nitrogen-doped TiO_2_ crystal plane is stronger than that on fluorine-doped TiO_2_ crystal plane. However, when nitrogen-doped TiO_2_ crystal plane reacts with SO_2_ and SOF_2_, the adsorption energy, charge transfer amount and adsorption distance have little difference, and the macro-gas-sensing characteristics all show the decrease of impedance. Therefore, nitrogen-doped TiO_2_ cannot effectively distinguish SO_2_ and SOF_2_. When fluorine-doped TiO_2_ crystal plane react with SO_2_, SO_2_ molecule obtains electrons, and fluorine-doped TiO_2_ gas sensor macro-gas-sensing characteristics show the increase of impedance. When fluorine-doped TiO_2_ crystal plane adsorb SOF_2_, SOF_2_ molecule loses electrons, and fluorine-doped TiO_2_ gas sensor macro-gas-sensing characteristics show the decrease of impedance. Although the adsorption of SO_2_ and SOF_2_ on fluorine-doped TiO_2_ crystal plane is weaker than that of nitrogen-doped TiO_2_ crystal plane, fluorine-doped TiO_2_ can effectively distinguish SO_2_ and SOF_2_.

## 4. Conclusions

In this paper, one O atom of the anatase TiO_2_ (101) perfect crystal plane is replaced by one F atom to obtain a fluorine-doped anatase TiO_2_ (101) perfect crystal plane model. H_2_S, SO_2_, SOF_2_ and SO_2_F_2_ of SF_6_ decomposition components adsorb on the F-doped TiO_2_ crystal plane in different ways to obtain different adsorption systems, which were then optimized. The adsorption mechanism is obtained by the optimized adsorption parameters. The adsorption results were compared and analyzed with intrinsic and nitrogen doping, with the effects of different doping on adsorption parameters studied. Through the above analysis, the following conclusions are drawn:

(1) Four kinds of gas molecules are close to the crystal plane, which cause gas molecules to be adsorbed on the crystal plane more easily. The adsorption ability of four kinds of gas molecules onto F-doped TiO_2_ crystal plane is: H_2_S > SO_2_ > SOF_2_ > SO_2_F_2_.

(2) The adsorption capacity of TiO_2_ to SO_2_, SOF_2_ and SO_2_F_2_ is obviously enhanced after fluorine doping, and the degree of the adsorption to SO_2_ gas molecules has reached the chemical adsorption.

(3) Based on the change of resistance of fluorine-doped TiO_2_ sensors on the macro level can effectively distinguish SO_2_ and SOF_2_ from theoretical analysis, even though the adsorption of SF_6_ decomposition components onto nitrogen-doped TiO_2_ crystal plane is stronger than that on fluorine-doped TiO_2_ crystal plane.

## Figures and Tables

**Table 1:** Adsorption parameters of four gas molecules on the perfect crystal plane of F-doped TiO_2_.

Adsorption System	Adsorption Structure	Adsorption Energy E_a_ (eV)	Charge transfer Amount Q_t_ (e)	Adsorption Distance (Å)
SO_2_-S-TiO_2_	a	−0.173	−0.013	2.847
SO_2_-O-TiO_2_	b	−0.132	0	3.229
SO_2_-2O-TiO_2_	c	−0.617	−0.12	2.111
H_2_S-S-TiO_2_	d	−0.209	0.008	2.835
H_2_S-H-TiO_2_	e	−0.837	0.267	2.714
H_2_S-2H-TiO_2_	f	−0.836	0.266	2.717
SOF_2_-S-TiO_2_	g	−0.254	−0.017	2.749
SOF_2_-O-TiO_2_	h	−0.412	0.098	2.438
SOF_2_-F-TiO_2_	i	−0.534	0.038	2.425
SOF_2_-2F-TiO_2_	j	−0.423	0.098	2.424
SO_2_F_2_-O-TiO_2_	k	−0.044	0	3.091
SO_2_F_2_-F-TiO_2_	l	−0.051	−0.002	2.863
SO_2_F_2_-2O-TiO_2_	m	−0.398	0.046	2.652
SO_2_F_2_-2F-TiO_2_	n	−0.198	−0.003	2.833
